# Supplementary material for: A Multiparametric MR-Based RadioFusionOmics Model with Robust Capabilities of Differentiating Glioblastoma Multiforme from Solitary Brain Metastasis
Source: Cancers (Basel). 2021 Nov 18;13(22):5793. doi: 10.3390/cancers13225793 (PMC8616314; doi:10.3390/cancers13225793)
Supplement: Supplementary file 1 [file cancers-13-05793-s001.zip › cancers-1469247-supplementary.pdf]

Supplementary Materials

A Multiparametric MR-Based RadioFusionOmics Model with Robust Capabilities of Differentiating Glioblastoma Multiforme from Solitary Brain Metastasis

Jialiang Wu, Fangrong Liang, Ruili Wei, Shengsheng Lai, Xiaofei Lv, Shiwei Luo, Zhe Wu, Huixian Chen, Wanli Zhang, Xiangling Zeng, Xianghua Ye, Yong Wu, Xinhua Wei, Xinqing Jiang, Xin Zhen and Ruimeng Yang

Table S1. Feature selection methods and classifiers.

**Feature selection methods(*n*=5)**	**double input symmetrical relevance (DISR), Joint Mutual Information (JMI), Spectral Feature Selection (SPEC), ll_121 , f_score**
**Classifiers(*n*=3)**	LogisticRegression(LR), Support vector machine(SVM), Gradient Boosting Decision Tree (GBDT)

Comparison between using majority voting and weighted fusion within the proposed RFO model:

Table S2. Performance comparison of majority voting and weighted fusion.

	**Independent testing set 1 (*n*=62)**				**Independent testing set 2 (*n*=61)**			
	**AUC**	**ACC**	**SEN**	**SPE**	**AUC**	**ACC**	**SEN**	**SPE**
**Proposed RFO model (majority voting)**	0.9246	0.8548	0.8561	0.8529	0.8592	0.8197	0.7083	0.8919
**Proposed RFO model (weighted fusion)**	0.9249	0.8548	0.8561	0.8529	0.8592	0.8361	0.7083	0.9189

Performance comparison on two VOIs (enhanced tumor (ET) and peritumoral edema (pTE)) using the excluded GBMs (*n* = 11) or SBMS (*n* = 19) as an independent testing cohort:

Table S3. Performance comparison using the excluded GBMs (*n* = 11) or SBMS (*n* = 19) as an independent testing cohort.

		**AUC**	**ACC**	**SEN**	**SPE**
**VOI_{ET}**	**Top-3 models' mean**	0.9569	0.8889	1.0000	0.6970
	**Proposed RFO model (weighted fusion)**	0.9665	0.9000	1.0000	0.7273
**VOI_{pTE}**	**Top-3 models' mean**	0.9107	0.8444	0.8772	0.7879
	**Proposed RFO model (weighted fusion)**	0.9330	0.8333	0.8947	0.7273
**VOI_{ET+pTE}**	**Top-3 models' mean**	0.9219	0.8000	0.8070	0.7879
	**Proposed RFO model (weighted fusion)**	0.9234	0.8333	0.8421	0.8182
